# Cytotoxic Oxidative Stress Effects of Neutrophil Extracellular Traps’ Components on Cattle Spermatozoa

**DOI:** 10.3390/antiox13060733

**Published:** 2024-06-17

**Authors:** Rodrigo Rivera-Concha, Marion León, Aurora Prado-Sanhueza, Raúl Sánchez, Anja Taubert, Carlos Hermosilla, Pamela Uribe, Fabiola Zambrano

**Affiliations:** 1 Center of Excellence in Translational Medicine—Scientific and Technological Bioresource Nucleus (CEMT—BIOREN), Faculty of Medicine, Universidad de La Frontera, Temuco 4780000, Chile; r.rivera07@ufromail.cl (R.R.-C.); m.leon06@ufromail.cl (M.L.); a.prado01@ufromail.cl (A.P.-S.); raul.sanchez@ufrontera.cl (R.S.); pamela.uribe@ufrontera.cl (P.U.); 2 Ph.D. Program in Medical Sciences, Faculty of Medicine, Universidad de La Frontera, Temuco 4780000, Chile; 3 Ph.D. Program in Morphological Sciences, Faculty of Medicine, Universidad de La Frontera, Temuco 4780000, Chile; 4 Department of Preclinical Sciences, Faculty of Medicine, Universidad de La Frontera, Temuco 4780000, Chile; 5 Institute of Parasitology, Justus Liebig University Giessen, 35392 Giessen, Germany; anja.taubert@vetmed.uni-giessen.de (A.T.); carlos.r.hermosilla@vetmed.uni-giessen.de (C.H.); 6 Department of Internal Medicine, Faculty of Medicine, Universidad de La Frontera, Temuco 4780000, Chile

**Keywords:** cattle, neutrophil extracellular traps, spermatozoa, oxidative stress

## Abstract

Bovine spermatozoa are highly susceptible to oxidative stress (OS), and it is known to affect their cellular functions. The main leukocyte producers of reactive oxygen species (ROS) in mammalian semen are polymorphonuclear neutrophils (PMN). PMN activation can result in the formation of neutrophil extracellular traps (NETs), which have been shown to affect the motility and function of spermatozoa. However, OS effects on bull spermatozoa derived from individual NETs components have not been investigated. The hypothesis of this study was that specific NETs components might generate OS on bull spermatozoa. Bovine sperm cells were incubated with five NETs-associated molecules, including 30 μg/mL histone 2A (H2A), neutrophil elastase (NE), 1 μg/mL myeloperoxidase (MPO), cathepsin G (Cat-G), and cathelicidin LL37 (LL-37), for a time course ranging from 15 to 240 min. Fluorescence microscopy was used to evaluate the coincubation of bovine PMN and sperm cells. Within 15 min, H2A, NE, and LL-37 caused membrane disruption, while MPO and Cat-G caused OS on bull spermatozoa after 1 h of coincubation. NET formation was observed within 15 min of coincubation in co-cultures of bovine PMN/sperm cells. This study is the first to report on the role of cytotoxic OS effects caused by NETs-derived components in bovine sperm in vitro.

## 1. Introduction

Reactive oxygen species (ROS), including hydrogen peroxide (H_2_O_2_), superoxide anion (O_2_^·−^), hydroxyl radical (^·^OH), and hypochlorite (ClO^−^) [1], are the primary cause of oxidative stress (OS) in cells [2]. Cells have several systems for controlling excess ROS, including superoxide dismutase, catalase, and the oxidized/reduced glutathione system (GSH/GSSG) [3]. ROS have diverse functions in cells when present in physiological concentrations. Thus, physiological levels of ROS play a crucial role in capacitation, hyperactivation, and acrosome reaction in spermatozoa [4]. However, when ROS generation exceeds the normal range and the systems cannot control it, cells experience OS [5]. In case of male gametocytes, OS leads to a loss of motility [6], DNA fragmentation [7], sperm membrane disruption, and ultimately apoptosis [8] in human and bovine sperm cells. Additionally, in bovines, spermatozoa exposed to OS excess can lead to DNA damage and subsequent developmental abnormalities in embryos [9].

Mammalian spermatozoa are highly susceptible to OS due to their transcriptionally silent nature and limited cytoplasmic volume, which reduce the number of ROS-controlling systems in these cells [10]. Additionally, the plasma membrane contains a high concentration of polyunsaturated fatty acids (PUFAs), such as docosapentaenoic and docosahexaenoic acids, making them highly vulnerable to lipoperoxidation [11]. The mitochondria is the main source of internal ROS, but in the case of spermatozoa in seminal fluid, leukocytes such as polymorphonuclear neutrophils (PMN) are the main external sources of ROS [1,12].

PMN are the initial leukocyte population to be recruited to any inflammation or infection site [13]. Activated PMN display various effector mechanisms, including phagocytosis, degranulation of microbial compounds into the extracellular medium, and release of neutrophil extracellular traps (NETs) [14]. These NETs are capable of killing fungi [15], parasites [16], and bacteria [14]. However, in cases of excess or inadequate removal of NETs, these extracellular structures are known to be involved in autoimmune, metabolic, and inflammatory disorders [17], becoming a “double-edged sword”. Decondensed DNA is the main component of extruded mammalian NETs. Citrunillated histones make up about 70% of the total protein of these DNA fibers and knowing to have cytotoxic and pro-inflammatory functions [18]. Other proteins, mostly originating from the cytoplasmic granules of PMN, such as myeloperoxidase (MPO), neutrophil elastase (NE), cathepsin G (CatG), cathelicidin LL-37 (LL-37), and calprotectin, among others, have also been identified as potent pro-inflammatory molecules [19]. Correspondingly, research has documented that released NETs can cause cytotoxic OS effects on exposed cells such as human enterocyte-like cells [20], mice epithelial cells, human endothelial cells [21], macrophages, and dendritic cells (DC) [22]. In bovines, the presence of PMN in the female reproductive tract (FRT) has been linked to infertility [23] and impairing fertility in artificial insemination (AI) [24].

NETs have also been found to entrap human sperm cells, thereby reducing their motility [25]. NETs within FRT have also been reported in equines [26], swine [27], and bovines [28]. Several NETs-associated proteins have been found to have adverse effects on bovine spermatozoa, resulting in plasma membrane disruption, acrosome damage, and lipoperoxidation [29]. However, the OS generated by these NETs components on spermatozoa has been scarcely reported [30]. Therefore, the aim of this study was to assess whether NETs-derived components might generate cytotoxic OS on exposed bull spermatozoa. To achieve this aim, the effects of five NETs-derived proteins on bovine spermatozoa at different concentrations and incubation times were assessed by measuring mitochondrial O_2_^·−^ generation, intracellular O_2_^·−^ generation, and lipoperoxidation. The present results will provide novel data on NETs-mediated OS effects on bovine sperm, thereby helping to better understand the crucial role of NETosis not only in domestic animals’ reproductive issues, but also in human reproductive disorders.

## 2. Materials and Methods

### 2.1. Ethical Declaration, Site, and Reagents

All the presented experiments and protocols were approved by the Universidad de La Frontera (UFRO) Scientific Ethics Committee, Temuco, Chile, with authorization code 120_20 and were performed in accordance with the current Chilean Statute N° 20,380 on “Protection of Animals”. All experiments were performed at the Center of Excellence in Translational Medicine—Scientific and Technological Bioresource Nucleus (CEMT—BIOREN), Faculty of Medicine, UFRO, Temuco, Chile. All reagents were from Sigma-Aldrich (St. Louis, MO, USA) unless indicated otherwise.

### 2.2. Sperm Selection

Bovine sperm cryopreserved in liquid nitrogen were thawed at 37 °C for 1 min and then separated using a Bovipure^®^ density-gradient kit (Nidacon, Mölndal, Sweden) with centrifugation at 600× *g* for 5 min; washed two times with 800 μL of Sperm-Talp^®^ medium according to the method described by Bavister and Yanagimachi [31], with some modifications [32]; centrifuged at 300× *g* for 4 min; and finally re-suspended in 200 μL of Sperm-Talp^®^ medium.

### 2.3. PMN Isolation

Bovine PMN were isolated from the peripheral blood of healthy dairy cows according to the method described by Roth and Kaeberle [33], with some modifications [34]. Briefly, peripheral blood was extracted from the jugular vein with a Vacutainer^®^ system in tubes with EDTA to avoid coagulation (Becton Dickinson and Company, BD Biosciences, San Jose, CA, USA). Then, 20 mL of blood was diluted in 20 mL sterile PBS supplemented with 0.2% EDTA. Then, this dilution was placed on a Hystopaque^®^-1077 separation solution (Sigma-Aldrich, St. Louis, MO, USA) and centrifuged at 800× *g* for 45 min. The supernatant was discarded, and erythrocytes were lysed for 1 min with 20 mL of lysis buffer. The tonicity was restored with a hypertonic solution, centrifuged at 600× *g* for 10 min, and the supernatant was discarded. The pellet was washed twice with 40 mL of Hank’s Balanced Salt Solution (HBSS) (Sigma-Aldrich, St. Louis, MO, USA), centrifuged again at 600× *g* for 10 min, and finally, the pellet was re-suspended in 3 mL of HBSS. The viability and purity of isolated bovine PMN were analyzed by exclusion with commercial trypan blue^®^ test in a Countess 3 FL system (Invitrogen, Life Technologies Corporation, Bothell, WA, USA).

### 2.4. DNA Staining

DNA staining was performed as described by Rivera-Concha et al. [35]. Briefly, PMN were incubated with bovine sperm at a 1:3 ratio in a time course of 15 to 240 min at 37 °C in 5% CO_2_ atmosphere. Cells were fixed with p-formaldehyde at 4% for 15 min, washed with sterile PBS, incubated with Hoechst 33342 (Invitrogen, Thermo Fisher Scientific, Waltham, MA, USA) for 15 min in a 1:2000 dilution in sterile PBS, washed with PBS. Then, the samples were incubated with Sytox Orange^®^ (Invitrogen, Thermo Fisher Scientific, Waltham, MA, USA) in a 1:2000 dilution in PBS for 15 min at room temperature (RT), washed with sterile PBS, and mounted with Fluoromount-G^TM^ mounting medium (Invitrogen, Thermo Fisher Scientific, Waltham, MA, USA) for later visualization in TissueFAXS i Plus Cytometry ^®^ (Tissue Gnostics, Vienna, Austria).

### 2.5. Sperm Treatments

In this experimental setting, sperm cells from three donors (*n* = 3) were incubated with different NET-derived components separately: 30 μg/mL histone H2A (H2A) and neutrophil elastase (NE), 1 μg/mL myeloperoxidase (MPO), cathepsin G (CatG), and cathelicidin LL-37 (LL-37), respectively, at 37 °C and 5% CO_2_ in a time course from 15 up to 240 min. Sperm cells from bulls (*n* = 3) incubated at 37 °C and 5% CO_2_ in a time course from 15 up to 240 min without adding a NET component were used as control. The concentration of each protein was determined according to Moya et al. [29]. A sperm concentration of 7.5 × 10^5^ vital spermatozoa suspended in sterile 400 µL of Sperm-Talp^®^ medium was used for the following sperm evaluation criteria: lipid peroxidation, mitochondrial O_2_^·−^ generation, and intracellular O_2_^·−^ generation.

### 2.6. Lipoperoxidation

Lipoperoxidation was determined using the BODIPY-C11^®^ probe (Invitrogen, Thermo Fisher Scientific, Waltham, MA, USA) at a final concentration of 5.8 μM according to Koppers et al. [1]. The sperm suspension was incubated at 37 °C for 30 min, then centrifuged at 300× *g* for 5 min. The pellet was re-suspended in 200 μL of Sperm-Talp^®^ medium and finally evaluated by flow cytometry analysis (FACScanto II^®^, Becton Dickinson and Company, BD Biosciences, San Jose, CA, USA).

### 2.7. Intracellular O_2_^·−^ Production

The intracellular O_2_^·−^ production was determined by incubating bovine spermatozoa with 5 μM hydroethidine (DHE) (Invitrogen, Thermo Fisher Scientific, Waltham, MA, USA) and 0.5 μM SYTOX^®^ Green (Molecular Probes, Eugene, OR, USA) for 30 min at 37 °C in the dark according to Koppers et al. [1], then centrifuging it at 300× *g* for 5 min. The pellet was re-suspended in 200 μL of Sperm-Talp^®^ medium and finally evaluated by flow cytometry analysis.

### 2.8. Mitochondrial O_2_^·−^ Production

The mitochondrial O_2_^·−^ production was determined by incubating the spermatozoa with 2 μM MitoSOX^TM^ red (Invitrogen, Thermo Fisher Scientific, Waltham, MA, USA) and 0.5 μM SYTOX^®^ Green (Molecular Probes, Eugene, OR) for 30 min at 37 °C in the dark according to Koppers et al. [1], then centrifuging it at 300× *g* for 5 min. The pellet was re-suspended in 200 μL of Sperm-Talp^®^ medium and finally evaluated by flow cytometry analysis.

### 2.9. Flow Cytometry Analysis

All flow cytometry-based sperm analyses were performed in a FACSCanto II^®^ flow cytometer (Becton Dickinson and Company, BD Biosciences, San Jose, CA, USA). The acquisition and analysis of samples were carried out using FACSDiva^TM^ v. 6.1.3 (BD Biosciences, San Jose, CA, USA) and data of 1 × 10^4^ sperm cell events were recorded. Data from three independent experiments were used. Manual gating was performed for the sperm population from the forward-scatter channel (FSC) versus the side-scatter channel (SSC). Autofluorescence controls without fluorophores were used as positive and negative controls to compensate for the use of fluorophores according to the method described by Zambrano et al. [25], with some modifications [29].

### 2.10. Statistical Analysis

Independent experiments were performed at least three times with different semen samples from the same bull. The results were presented as mean ± standard deviation (SD). The GraphPad Prism^®^ software v. 10.1.1 was used for the statistical analyses. The D’Agostino–Pearson K^2^ and Shapiro–Wilk tests were used to evaluate the normal distribution. If they failed to pass the normality test, the values were transformed into their arcsine value. Student’s *t*-test was used to evaluate significant differences between each time slot. A level of *p* < 0.05 was considered significant.

## 3. Results

### 3.1. Sperm-Triggered NETs and NETs Phenotypes

**Figure 1:**
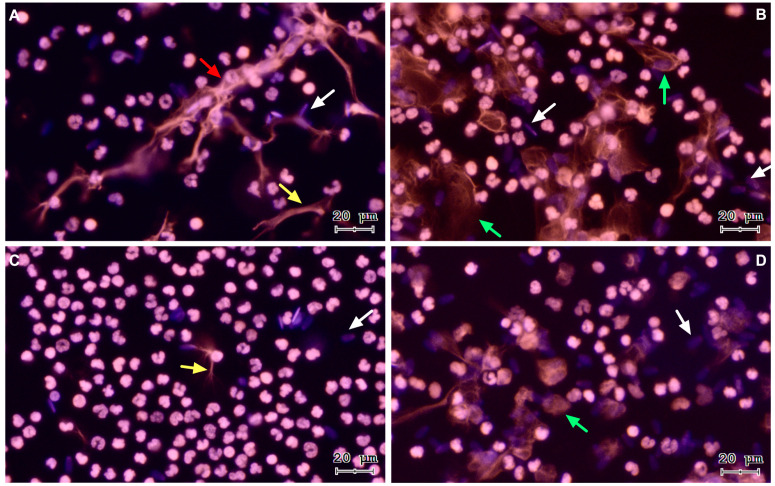
Representative images of DNA staining via Sytox^®^ Orange and Hoechst 33342 at different time points of PMN and bovine spermatozoa coincubation, (**A**) 15 min, (**B**) 60 min, (**C**) 120 min, and (**D**) 240 min. White arrows: sperm heads, yellow arrow: *spr*NET, green arrows: *diff*NETs, red arrow: *agg*NETs. All images were acquired using 20× objective.

**Figure 2:**
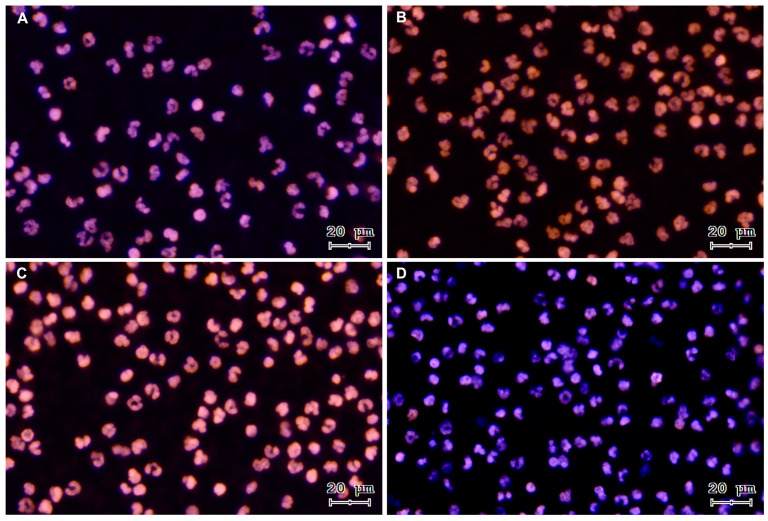
Representative images of DNA staining with Sytox^®^ Orange and Hoechst 33342 from a control time course of PMN incubated alone: (**A**) 15 min, (**B**) 60 min, (**C**) 120 min, and (**D**) 240 min. All images were acquired using 20× objective.

To verify sperm-triggered NETosis, motile bovine spermatozoa were incubated with PMN. Figure 1 shows representative images of a time course from a co-culture of isolated PMN and bovine spermatozoa. The three NETs phenotypes became apparent after PMN exposure to bovine sperm: aggregated NETs (*agg*NETs) were extruded from different PMN forming large extracellular aggregates (1A); spread NETs (*spr*NETs), which appeared as long DNA fibers extruding from one point of a single PMN (1A, 1C); and diffuse NETs (*diff*NETs), appearing as a “cloud of DNA” (1B, 1D). Bovine PMN incubated alone in medium failed to release NETs up to 4 h of incubation (Figure 2A–D).

### 3.2. CatG Treatment

**Figure 3:**
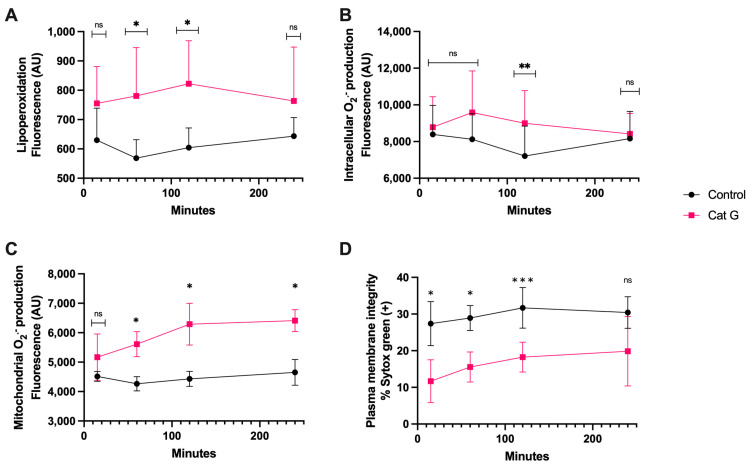
Time course for the coincubation of 1 μg/mL CatG with bovine spermatozoa. (**A**) Lipoperoxidation, (**B**) intracellular O_2_^·−^ production, (**C**) mitochondrial O_2_^·−^ production, and (**D**) plasma membrane disruption. The results are shown as the mean ± standard deviation. The asterisks (*, **, ***) indicate significant differences between the groups (*p* < 0.05, *p* < 0.01, and *p* < 0.001, respectively) based on three biological replicates. ns, not significant.

The treatment with CatG generated lipoperoxidation (Figure 3A) at 60 and 120 min of incubation and generation of intracellular O_2_^·−^ occurred at 120 min, which was significantly different than the results observed for the controls (Figure 3B). The generation of mitochondrial O_2_^·−^, however, was detectable at 60 min and continued up to 240 min (*p* < 0.05) (Figure 3C). Finally, after as little as 15 min of sperm exposure to CatG, there was a significant increase in plasma membrane disruption compared to the control, and this remained detectable until 120 min of incubation (*p* < 0.001) (Figure 3D).

### 3.3. H2A Treatment

**Figure 4:**
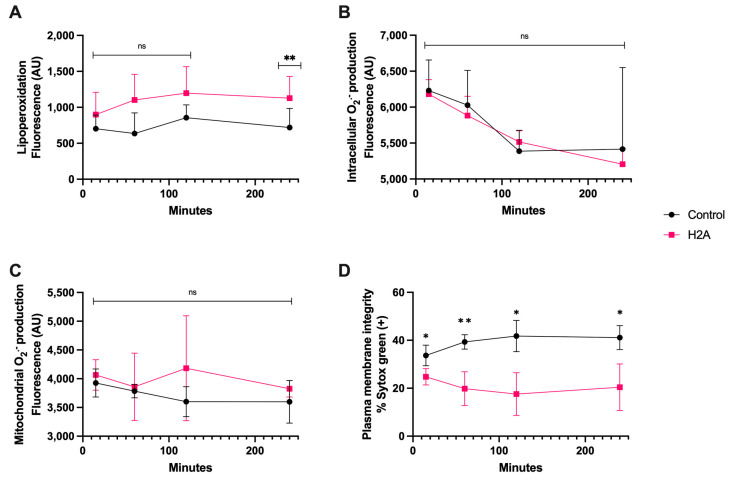
Time course for the coincubation of 30 μg/mL H2A with bovine spermatozoa. (**A**) Lipoperoxidation, (**B**) intracellular O_2_^·−^ production, (**C**) mitochondrial O_2_^·−^ production, and (**D**) plasma membrane disruption. The results are shown as the mean ± standard deviation. The asterisks (*, **) indicate significant differences between the groups (*p* < 0.05 and *p* < 0.01, respectively) based on three biological replicates.

Regarding the H2A treatment, it generated detectable lipoperoxidation on spermatozoa at only 240 min of coincubation, (Figure 4A), and there were no statistically significant differences in intracellular and mitochondrial O_2_^·−^ generation (Figure 4B,C). Still, there was plasma membrane disruption that was statistically different from the control from 15 min of incubation up to 240 min (*p* < 0.05), demonstrating the cytotoxic properties of H2A (Figure 4D), and that the H2A-dependent cytotoxic properties of sperm were not linked to OS.

### 3.4. LL-37 Treatment

**Figure 5:**
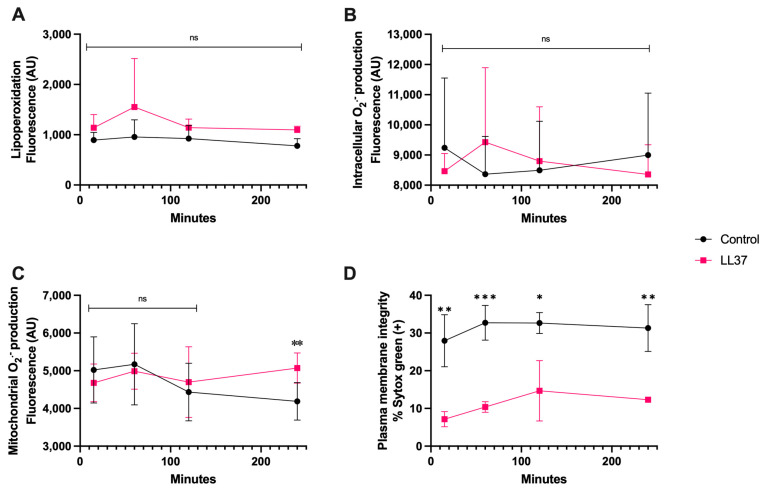
Time course for the coincubation of 1 μg/mL LL-37 with bovine spermatozoa. (**A**) Lipoperoxidation, (**B**) intracellular O_2_^·−^ production, (**C**) mitochondrial O_2_^·−^ production, and (**D**) plasma membrane disruption. The results are shown as the mean ± standard deviation. The asterisks (*, **, ***) indicate significant differences between the groups (*p* < 0.05, *p* < 0.01, and *p* < 0.001, respectively) based on three biological replicates.

In the case of coincubation of spermatozoa with LL-37 (1 μg/mL), Figure 5A,B show that at this concentration there was neither lipoperoxidation nor generation of intracellular O_2_^·−^, nonetheless, there was a significant increase in mitochondrial O_2_^·−^ generation at 240 min of coincubation (*p* < 0.01) (Figure 5C), and plasma membrane disruption already significantly differed from that of the control from 15 min up to 120 min (*p* < 0.01) (Figure 5D).

### 3.5. MPO Treatment

**Figure 6:**
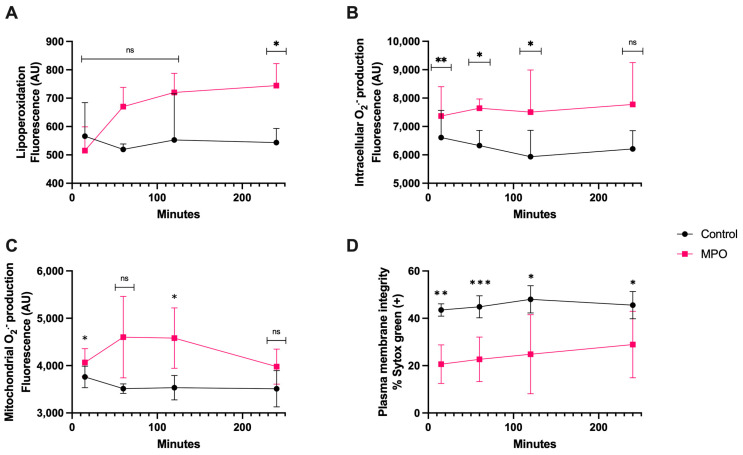
Time course for the coincubation of 1 μg/mL MPO with bovine spermatozoa. (**A**) Lipoperoxidation, (**B**) intracellular O_2_^·−^ production, (**C**) mitochondrial O_2_^·−^ production, and (**D**) plasma membrane disruption. The results are shown as the mean ± standard deviation. The asterisks (*, **, ***) indicate significant differences between the groups (*p* < 0.05, *p* < 0.01, and *p* < 0.001, respectively) based on three biological replicates.

MPO treatment generated significant lipoperoxidation (*p* < 0.05) only after 4 h of coincubation (Figure 6A). The intracellular O_2_^·−^ generation was greater than control as early as 15 min (*p* < 0.01) and it was maintained for up to 2 h (*p* < 0.05) (Figure 6B). The mitochondrial O_2_^·−^ production was significantly different than that of the control at 15 min and 2 h (*p* < 0.05). There was no difference at 4 h (Figure 6C). The plasma membrane disruption compared with that of the control showed a significantly higher increase at 15 min (*p* < 0.01) and 1 h (*p* < 0.0001) than at 2 and 4 h of coincubation (*p* < 0.05) (Figure 6D).

### 3.6. NE Treatment

**Figure 7:**
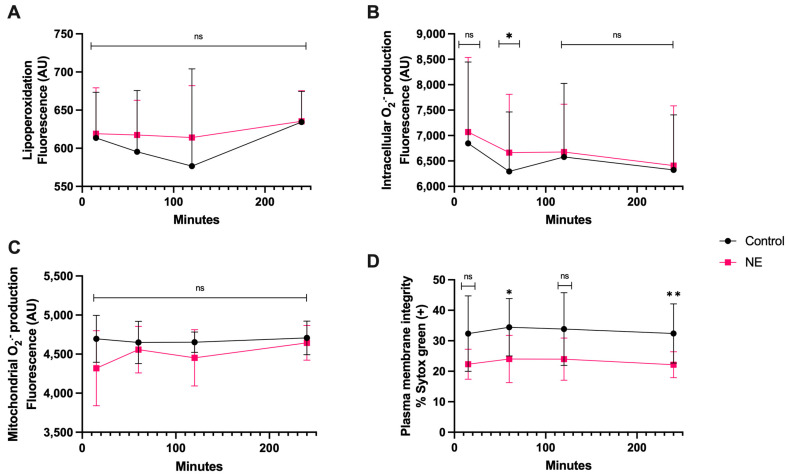
Time course for the coincubation of 30 μg/mL NE with bovine spermatozoa. (**A**) Lipoperoxidation, (**B**) intracellular O_2_^·−^ production, (**C**) mitochondrial O_2_^·−^ production, and (**D**) plasma membrane disruption. The results are shown as the mean ± standard deviation. The asterisks (*, **) indicate significant differences between the groups (*p* < 0.05 and *p* < 0.01, respectively) based on three biological replicates.

There was no significant statistical difference in lipoperoxidation and mitochondrial O_2_^·−^ generation between the co-culture with NE and spermatozoa compared to the control during this time course (Figure 7A,C). There was only a significant difference in intracellular O_2_^·−^ production at 1 h of coincubation compared to the control (*p* < 0.05). Plasma membrane disruption appeared significant at 1 h (*p* < 0.05) and 4 h of coincubation, with a higher percentage of disruption at 4 h (*p* < 0.01) (Figure 7D).

## 4. Discussion

The first report of oxidative damage generated by extruded NETs was the article by Donis-Maturano et al. [22]. Former authors showed the effects of NETs-derived OS on immunocompetent cells, specifically macrophages and dendritic cells (DC). In humans, several lines of evidence indicate that OS and the presence of ROS are related to male infertility [36] and sperm motility [6]. Cytotoxic OS has also been linked to various health issues and immune disorders in dairy cattle [37] and infertility in male animals [38].

The present study demonstrates that bovine spermatozoa stimulate PMN to produce three distinct NETs phenotypes, a phenomenon that was previously reported for other mammalian species, including humans [25,39,40]. Our group has previously reported negative effects of NETs components on bovine spermatozoa, but the effects of OS have not been extensively studied [29].

CatG is known to generate mitochondrial OS and cytotoxic effects on spermatozoa of various host species. In addition, it has been reported that CatG expression is up-regulated in PMN of human patients with asthma [41] and is involved in inflammatory responses of chronic obstructive pulmonary diseases [42]. In mares, NETs-derived CatG is associated with fibrosis in endometrium explants [43,44] thereby demonstrating pro-inflammatory properties. All these findings clearly demonstrate CatG-derived cytotoxic effects in these tissues, but none of them exhibit damage mainly caused by OS, and the present study is the first one to show adverse effects of CatG-derived OS on exposed mammalian sperm cells.

For spermatozoa exposed to H2A derived from NETs, we observed a disruption in the plasma membrane after only 15 min of incubation, as well as lipoperoxidation after 240 min, which was consistent with previous findings [29]. In this scenario, it has been reported that H2A caused damage not only to human primary epithelial and endothelial cells [21] but also to murine intestinal epithelial cells [45]. Furthermore, cell exposure to H2A resulted in the cell death of macrophages and synoviocytes in human patients with rheumatoid arthritis [46]. Additionally, high concentrations of extracellular histones in blood plasma were found to be correlated with the severity of COVID-19 [47]. H2A mainly acts by disrupting the plasma membrane and mitochondrial outer membrane permeability [48], which is consistent with our results.

In our study, we observed MPO-derived intracellular and mitochondrial O_2_^·−^ generation, which caused cytotoxic OS in exposed bovine spermatozoa. It has been reported that the in vivo MPO concentration can reach up to 1–2 mM, thereby causing damage to invasive pathogens [49] in surrounding tissues [50,51]. Meanwhile, in our study, the concentration used was 12 nM (1 μg/mL). In a murine model, NETs and eosinophil extracellular traps (EETs) eliminated the third-stage larvae of *Strongyloides ratti* in an MPO-dependent manner [52]. MPO has also been observed in bovine macrophage extracellular traps released in response to the presence of the abortive parasite *Neospora caninum* [53]. In humans, the MPO–DNA complex was found to be elevated in the blood of venous thromboembolism patients for up to one year [54]. These findings indicate the importance of MPO in extracellular traps (ETs) causing oxidative damage in ETs-entrapped cells or pathogens. This could be crucial to improving our understanding of the molecular mechanisms involved in the detrimental effects of NETs on mammalian spermatozoa.

LL-37 exerts a cytotoxic effect through plasma membrane disruption and does not exhibit any observable OS. As one of many of so-called antimicrobial peptides (AMPs) [55], thus far, it is the only component of NETs to have a proven spermicidal effect in both mice and humans [56]. This spermicidal effect is partially because LL-37 has the capacity to bind acidic sulfogalactosylglycerolipid (SGG) on the sperm cell plasma membrane [56]. Additionally, it is overexpressed in activated PMN of patients with systemic lupus erythematosus (SLE), contributing to vessel endothelium damage generated by released NETs in this autoimmune disease [57]. This opens up questions on defensins, the other family of AMPs present in mammals, and their presence and role in spermatozoa-activated NETs.

The results indicate that NE has a cytotoxic effect resulting in minor intracellular O_2_^·−^ generation after 60 min. NE, similar to CatG, has been implicated in the pathophysiology of inflammatory lung diseases [58]. However, the presence of elastase has been linked to higher ROS levels in human spermatozoa [59] and may contribute to decreased sperm quality during infections [60].

Despite the fact that the present work has certain limitations, i.e., it was conducted in vitro and/or without the presence seminal plasma, it helps to decipher NET-derived spermicidal OS effects in further detail. Thus, present results should not be extrapolated to an in vivo situation without additional research. Nonetheless, NETs-associated molecules might have synergistic adverse OS effects, particularly on the membrane integrity of bovine spermatozoa. As sperm cells used in our experiments were deprived of seminal plasma (SP) which naturally possess antioxidant systems [61] future in vitro sperm-NETs experiments with presence of SP are planned. As thawed cryopreserved bull spermatozoa used in AI contain extenders as well as traces of SP, and SP alone is capable of triggering bovine PMN to release NETs [62], we intend to address the possible antioxidant roles of SP/extenders in future OS experiments. An in vitro study on donkeys has shown that NETs release leads to extracellular H_2_O_2_ production in spermatozoa; however, this is the only other known report in the literature regarding NETs causing OS in sperm cells [30].

## 5. Conclusions

In conclusion, CatG and MPO primarily exert their cytotoxic effects by generating intracellular and mitochondrial O_2_^·−^, which in turn provoke OS in bovine spermatozoa, while NE, LL-37, and H2A directly affect the plasma membrane through different mechanisms. This is the first report of intracellular and mitochondrial OS generated by NETs components on mammalian spermatozoa. These results show the ability of sperm cells to trigger NETs, and sperm motility being a trigger for this process in cattle in vitro raises questions about the physiological role of NETs activated by spermatozoa in the bovine FRT in vivo. Therefore, further research is needed to elucidate the pathophysiology of this process.

## Data Availability

The raw data supporting the conclusions of this article will be made available by the authors on reasonable request.
